# Daily hot‐water immersion preserves altitude‐induced haemoglobin mass expansion following descent independent of erythropoietin

**DOI:** 10.1113/EP093944

**Published:** 2026-05-07

**Authors:** Elliott J. Jenkins, Jodie L. Koep, Andrew J. M. Douglas, Lauren E. Maier, Connor A. Howe, Sarah Sheitelman, Liam D. Corr, Christoph Siebenmann, Michael G. Hughes, Joshua C. Tremblay, Philip N. Ainslie, Travis D. Gibbons, Mike Stembridge

**Affiliations:** ^1^ Cardiff School of Sport and Health Sciences Cardiff Metropolitan University Cardiff UK; ^2^ Centre for Heart, Lung and Vascular Health, School of Health and Exercise Sciences University of British Columbia Okanagan Campus Kelowna Canada; ^3^ Institute of Sport Science University of Innsbruck Innsbruck Austria; ^4^ Department of Biological Sciences Northern Arizona University Flagstaff Arizona USA; ^5^ Institute of Mountain Emergency Medicine, EURAC Research Bolzano Italy; ^6^ Hochgebirgsklinik Davos, Medicine Campus Davos Davos Switzerland

**Keywords:** altitude training, haemoglobin mass, heat acclimation

## Abstract

High‐altitude exposure increases haemoglobin mass (Hb_mass_), a key determinant of arterial oxygen‐carrying capacity, but following descent this adaptation can regress toward baseline within 7 days. Long‐term heat acclimation has emerged as an alternative stimulus for Hb_mass_ expansion; however, whether post‐altitude passive‐heat exposure can preserve altitude‐induced increases in Hb_mass_ remains unclear. Furthermore, the extent to which any preservation of Hb_mass_ is supported by sustained erythropoietin (EPO) production, and whether this support is mediated by plasma volume expansion or acute alterations in renal haemodynamics, has yet to be explored. Twenty‐one healthy adults (8F) sojourned at 3800 m for 14 days and, following descent to 1250 m, were allocated to either hot‐water immersion (HWI; 45 min at 40°C for 4 days; *n *= 11) or control (CON; *n *= 10). Hb_mass_, intravascular volumes (carbon monoxide rebreathing) and circulating EPO were measured on high‐altitude day 1 (HA1), day 14 (HA14), and post‐descent day 5 (P5). Renal‐artery blood velocity (Doppler‐ultrasound) was measured before and after HWI on the day of descent. Hb_mass_ increased by 24 g [95% CI: 8, 40] across all participants during the altitude sojourn (*P *= 0.005). Following descent, Hb_mass_ decreased in CON (∆ = −18 g [95% CI: −36, 0], *P *= 0.045) but was maintained in HWI (∆ = +9 g [95% CI: −8, 26], *P *= 0.285). Circulating EPO declined after descent (*P *< 0.001) with no between‐condition difference at P5 (*P *= 0.239), despite a transient reduction in renal‐artery blood velocity following HWI (*P *= 0.025) and similar plasma volume expansion across groups (time: *P *< 0.001). Hot‐water immersion offers a practical, lower‐impact alternative to exercise‐heat methods for preserving altitude‐derived Hb_mass_ expansion, although the mechanisms underlying this response remain elusive.

## Abstract (highlights)

- **What is the central question of this study?**
  Can the high altitude‐induced increase in haemoglobin mass be preserved following return to low altitude by daily hot‐water immersion?
- **What is the main finding and its importance?**
  Across 5 days post‐descent, daily hot‐water immersion maintained haemoglobin mass, whereas it declined in controls. Plasma volume re‐expanded similarly across conditions, yielding a greater total blood volume with hot‐water immersion. Circulating erythropoietin concentrations decreased after descent in both groups, indicating preservation of haemoglobin mass without sustained EPO elevation. Passive heating via hot‐water immersion offers a practical, lower impact alternative to exercise‐heat methods for sustaining high‐altitude‐derived haematological benefits during deacclimitisation, extending the post‐altitude window for improved endurance performance.

## 1 INTRODUCTION

Altitude training is widely adopted by endurance athletes to promote physiological adaptations that may enhance sea‐level performance (Baranauskas et al., 2021; Levine & Stray‐Gundersen, 1997). Of these adaptations, the most ergogenic is an increase in total haemoglobin mass (Hb_mass_), owing to its direct influence on arterial oxygen‐carrying capacity and, consequently, maximal oxygen consumption (${\dot{V}}_{O_{2}\max}$) (Schmidt & Prommer, 2010). Increases in Hb_mass_ at altitude are driven by hypoxia‐induced erythropoiesis (Clause et al., 1996; Rodríguez et al., 2000), characterised by a transient rise in circulating erythropoietin (EPO) during the first 1–3 days of exposure before settling at modestly elevated levels (Płoszczyca et al., 2018). Following descent to sea level, withdrawal of the hypoxic stimulus results in a rapid decline in circulating EPO concentrations, in some cases falling below pre‐ascent values (Saugy et al., 2022). As a result, Hb_mass_, haematocrit (Hct), and reticulocyte count typically return to baseline within 7–14 days (Klein et al., 2021; Mairbäurl, 2018; Merino, 1950; Ryan et al., 2014). This transient response creates a logistical challenge for athletes, who must carefully align altitude camps with competition schedules to preserve the performance benefits of increased Hb_mass_ (Chapman et al., 2014).

Accumulating evidence has highlighted the potential for heat stress to act as an alternative environmental stimulus for haematological adaptation. Long‐term heat acclimation, achieved through repeated heat exposures (commonly ∼5 sessions per week for ≥5 weeks), has been shown to increase Hb_mass_ and ${\dot{V}}_{O_{2}\max}$ (reviewed in Jenkins et al., 2025a, 2025b; Lundby et al., 2023; Oberholzer et al., 2019; Rønnestad et al., 2021, 2022a, 2022b). Although the precise mechanisms remain uncertain (Jenkins et al., 2025a), evidence for a sustained role of circulating EPO is limited; a single hot‐water immersion (HWI) increased circulating EPO in females but not males in the only study to assess this directly (DiMarco et al., 2024). These combined observations suggest that heat exposure may provide a means of sustaining altitude‐induced haematological adaptations. Indeed, following a 3‐week altitude camp (2100 m) in elite cyclists, Rønnestad et al. (2024) reported that incorporating three heat‐training sessions per week during a 3.5‐week post‐altitude period preserved the altitude‐induced increase in Hb_mass_, whereas it declined in a control group. This approach relied on *active* heat training which, although effective, may constrain the intensity or structure of usual training practices and increase cumulative physiological strain (Periard et al., 2021). Such demands may limit its feasibility for athletes aiming to restore high‐quality training after altitude exposure. *Passive* modes of heat acclimation, such as HWI, induce considerable thermal strain with minimal mechanical load, are straightforward to implement, and do not necessitate adjustments to normal training (Heathcote et al., 2018) – a notable advantage during the post‐altitude, pre‐competition period. While HWI can increase Hb_mass_ independently (Jenkins et al., 2025b), whether it can preserve altitude‐induced haematological adaptations is unknown. Establishing this is important, as HWI may offer a practical, low‐load alternative to *active* heat training for prolonging altitude‐derived haematological benefits.

Despite the efficacy reported by Rønnestad et al. (2024), the mechanisms through which heat might sustain post‐altitude Hb_mass_ are not well understood. In their active heat‐training model, they speculated that preserved Hb_mass_ might relate to changes in plasma volume (PV), attenuation of the usual post‐altitude fall in EPO, or cellular stress signalling involving heat‐shock and hypoxia‐inducible pathways, though none of these mechanisms were directly assessed. More broadly, heat‐induced haematological adaptation has been attributed to PV expansion lowering Hct and engaging a ‘critmeter’‐driven erythropoietic response (Donnelly, 2001; Dunn & Donnelly, 2007; Rønnestad et al., 2021), while acutely, the increase in EPO observed after a single HWI bout was linked to reduced renal blood flow and oxygenation during heat stress (DiMarco et al., 2024). Conversely, Carin et al. (2025) report that Hb_mass_ increases during altitude exposure (1850 m) can occur despite unchanged circulating EPO, indicating that EPO‐independent pathways may also contribute. Consequently, the mechanisms by which heat might maintain altitude‐induced Hb_mass_ increases remain unclear, underscoring the need to examine acute changes in PV, renal haemodynamics and circulating EPO in the immediate post‐altitude period.

Accordingly, the present study examined whether performing HWI across four consecutive days in the immediate post‐descent period could preserve the Hb_mass_ adaptations acquired at altitude. We hypothesised that HWI would attenuate the regression of Hb_mass_ compared with a control group, supported by sustained elevations in circulating EPO and PV expansion. To provide mechanistic insight, the acute responses of PV and renal artery blood velocity were assessed as potential modulators of circulating EPO during HWI.

## 2 METHODS

### 2.1 Ethical approval

Study procedures were approved by the University of British Columbia Ethics Committee (H22‐01091) and conformed to the *Declaration of Helsinki* (2013), excluding prior registration in a database. Written, informed consent was obtained from each participant prior to testing.

### 2.2 Study design

A parallel‐group, repeated‐measures design was used to compare physiological responses between participants allocated to post‐altitude HWI or control (CON). Participants were matched as closely as possible for age, height, body mass, and self‐reported weekly training volume before allocation to HWI or CON.

**FIGURE 1:**
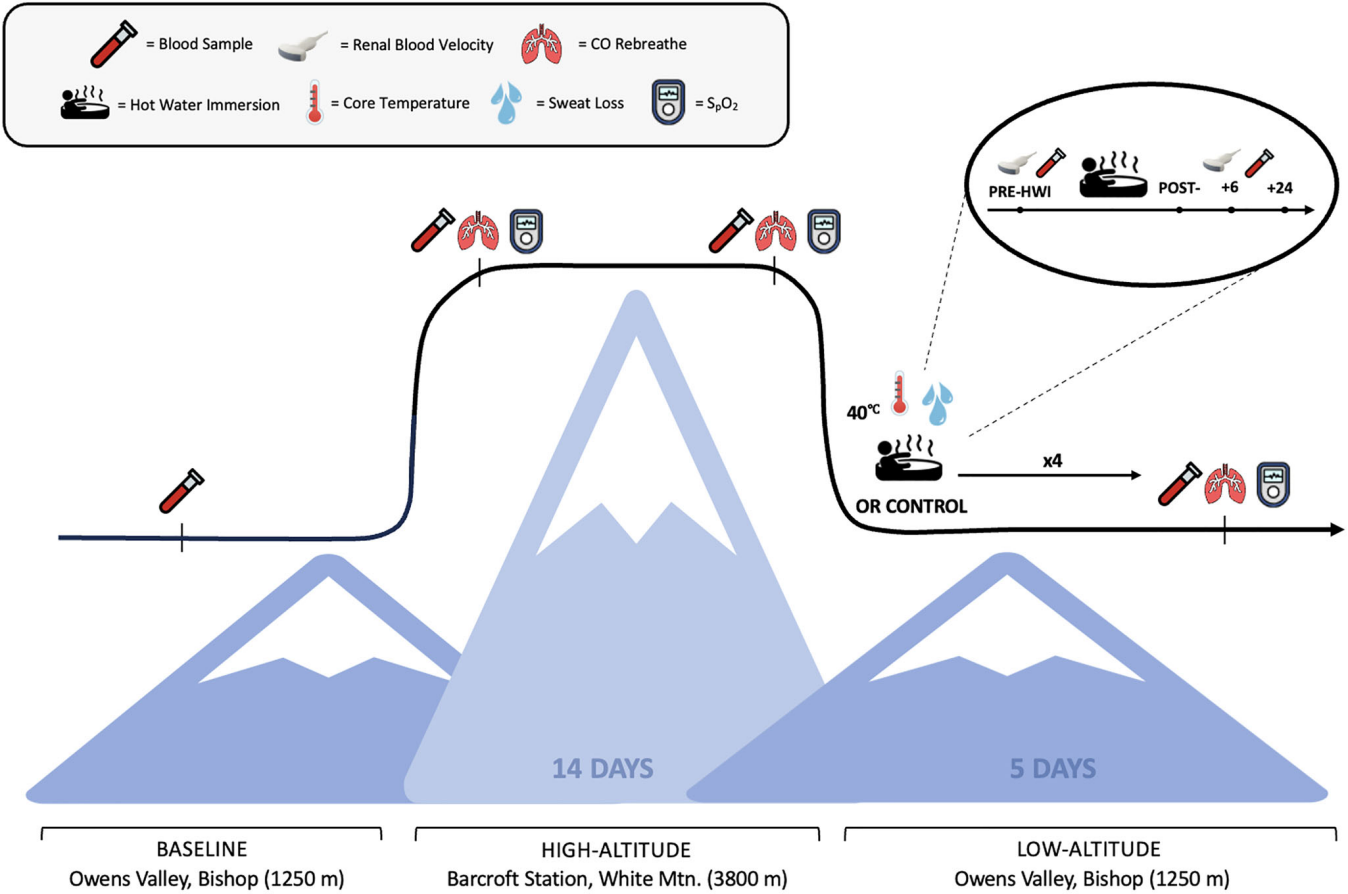
Study timeline and measurement schedule. Baseline blood sampling was performed at low altitude (Owens Valley, 1250 m) before a 14‐day sojourn at high altitude (Barcroft Station, 3800 m). On days 1 and 14 at high altitude, blood sampling and CO‐rebreathing were used to assess Hb_mass_, PV, RBCV and BV, with peripheral oxygen saturation recorded concurrently. After descent to 1250 m, participants were allocated to hot‐water immersion (HWI; 45 min at 40°C on four consecutive days; *n* = 11) or control (CON; *n* = 10). On the day of descent, an intensive 24‐h measurement cycle was completed with blood sampling (including EPO and [Hb]) and renal artery blood velocity (time‐averaged maximum; TAMAX) obtained Pre‐, Post‐, +6 h and +24 h relative to the first HWI/CON session. Thermal strain during HWI (tympanic temperature, sweat loss, thermal sensation/discomfort) was recorded. On post‐descent day 5, blood sampling and CO‐rebreathing were repeated.

An overview of the study timeline is presented in Figure 1. Baseline blood samples were collected at low altitude (Owens Valley, CA, USA; 1250 m) prior to ascent. Participants then completed a 14‐day sojourn at high altitude (Barcroft Station, White Mountain, CA, USA; 3800 m), with blood sampling and Hb_mass_ measurements performed on high‐altitude day 1 (HA1) and 14 (HA14). After descent back to 1250 m, acute responses to the thermal interventions were assessed over the first 24 h (post‐altitude day 1; P1), with measurements taken immediately pre‐ and post‐HWI (or time‐matched CON), and again at +6 h and +24 h after immersion (or time‐matched CON). The HWI group completed one immersion per day for the subsequent three days (4× immersion total), while CON participants underwent the deacclimation period without intervention. On post‐altitude day 5 (P5), all participants repeated the blood sampling and Hb_mass_ measurements to quantify haematological status at the end of the intervention.

### 2.3 Participants

Twenty‐one participants (13 males, 8 females) were recruited and allocated to either CON or HWI. The target sample size was guided by the feasibility constraints of the expedition and by precedent from similar altitude–heat interventions (Rønnestad et al., 2024). Given the typical error of Hb_mass_ measurements in our laboratory (1%), a sensitivity analysis (α = 0.05, power = 0.80) indicated that group sizes of 11 and 10 provided adequate power to detect between‐group differences in the change in Hb_mass_ of ∼2%. Participant characteristics are presented in Table 1. All participants were healthy, unacclimated to heat and drawn from a non‐athlete population with varied training backgrounds. The majority of participants were altitude‐naive; however, three participants resided at moderate altitude (∼2100 m; Flagstaff, AZ, USA) prior to the study, all of whom were allocated to CON. Before each measurement time point, participants were instructed to: (i) refrain from strenuous exercise and alcohol for 24 h; (ii) standardise caffeine intake; and (iii) maintain adequate hydration, as indicated by clear or pale urine colour. Menstrual cycle phase or contraceptive use was not controlled for in female participants due to logistical constraints inherent to expedition research.

**TABLE 1:** Participant characteristics for control (CON) and hot‐water immersion (HWI) groups.

	CON		HWI		*P*
	Mean	SD	Mean	SD	
*n*	10	—	11	—	—
Males	6	—	7	—	—
Females	4	—	4	—	—
Age (years)	27.3	4.6	27.9	6.2	0.799
Height (cm)	172.8	9.3	176.3	10.1	0.423
Mass (kg)	70.6	7.5	72.7	13.9	0.666
Lean body mass (kg)	52.8	8.6	55.4	10.8	0.551

*Note*: Data are means and SD. *P*‐values are from unpaired *t*‐tests comparing CON and HWI.

### 2.4 Experimental procedures

#### 2.4.1 Venous blood sampling

Venous blood samples were collected from the antecubital fossa at eight time points: pre‐ascent baseline (BL), HA1, HA14, P1 (pre‐, post‐, +6 h, +24 h) and P5. Samples for EPO analysis were drawn into 8.5 mL serum separator tubes (Becton‐Dickinson, Franklin Lakes, NJ, USA), inverted 5–6 times, allowed to clot for ≥30 min, and centrifuged at 1600 *g* for 10 min at room temperature. Serum was pipetted into Eppendorf tubes, snap‐frozen in liquid nitrogen, and subsequently stored at −80°C until analysis.

#### 2.4.2 Hb_mass_ and intravascular volumes

Hb_mass_ and intravascular volumes were assessed at HA1, HA14 and P5 using the CO‐rebreathing method with an automated system and software (Detalo Performance; Detalo Health, Birkerød, Denmark) as previously described (Jenkins et al., 2025b), with the modification that venous rather than capillary samples were obtained. Participants rested supine with feet elevated (∼30 cm) for 10 min before an initial venous blood sample (∼2 mL) was drawn. Hct was determined in quadruplicate following centrifugation at ∼10,000 g for 5 min (Haematospin 1400; Hawksley, Fishersgate (Sussex), UK) and read using a micro‐haematocrit reader (Hawksley). Haemoglobin concentration ([Hb]) and carboxyhaemoglobin (%COHb) were measured in duplicate using a blood gas analyser (ABL90; Radiometer, Copenhagen, Denmark). Participants then breathed 100% O_2_ for 1 min before rebreathing a CO bolus (1.0 mL kg^−^^1^ males, 0.8 mL kg^−^^1^ females) in a closed circuit for 6 min. A second venous blood sample was collected 4 min after the rebreathing phase to assess post‐rebreathing %COHb. Residual CO not absorbed was quantified by multiplying the CO concentration (parts per million, ppm) in the circuit by the total system volume (breathing apparatus + estimated pulmonary volume). The change in %COHb was then used to calculate Hb_mass_, accounting for the fraction of CO remaining in the rebreathing circuit at the end of the procedure. PV, red blood cell volume (RBCV) and total blood volume (BV) were subsequently derived from measurements of Hb_mass_ (typical error 1.0%), [Hb] and Hct (Burge & Skinner, 1995).

#### 2.4.3 Renal artery blood velocity

Renal artery blood velocity (time‐averaged maximum velocity; TAMAX) was measured via Doppler ultrasound (Vivid iq; GE Healthcare, Chicago, IL, USA) using a curved‐array transducer (4C‐RS^2^, GE Healthcare) as previously described (Chapman et al., 2020; DiMarco et al., 2024). Assessments were performed on P1 at four time points: pre‐HWI, post‐HWI, +6 h and +24 h (or equivalent CON time points). Renal artery blood velocity was assessed in the distal segment of the right renal artery via a coronal approach, with the participant lying in the left lateral recumbent position. A minimum of three consecutive cardiac cycles were acquired during a mid‐exhalation, non‐Valsalva breath hold to maximise image quality. The same artery was consistently measured in each participant and by the same sonographer (J.K.).

#### 2.4.4 Hot water immersion protocol

Participants assigned to the intervention group undertook four consecutive daily HWI sessions during the post‐altitude period (P1–P4). Each session consisted of 45 min seated upright in a tub heated to 40°C. To characterise the thermal strain, tympanic temperature (ThermoScan 7; Braun, Kronberg, Germany), sweat loss (change in body mass pre‐ to post‐immersion, adjusted for fluid intake), thermal sensation (1–13; unbearably cold–unbearably hot), and thermal discomfort (1–10; comfortable–extremely uncomfortable; both adapted from Gagge et al. (1967)) were recorded before, during and after each session. Participants were instructed to immerse to the neck but could reduce to chest level if required to complete the full session. Up to 500 mL of drinking water was permitted ad libitum during immersion.

### 2.5 EPO analysis

EPO concentration was measured using a Human EPO ELISA Kit (Invitrogen, Thermo Fisher Scientific, Waltham, MA, USA) with an assay sensitivity of 0.14 mIU mL^−^^1^ and a working range of 1.6–100 mIU mL^−^^1^. Serum samples were thawed fully and kept on ice until analysis. A 50 µL aliquot was loaded per well at a 1:2 dilution. Absorbance was read on a colorimetric microplate reader, and concentrations were derived from a standard curve generated with kit standards. All samples were assayed in duplicate, and the mean value was used for analysis. Samples with both replicates below the detection limit were treated as non‐detects and excluded. The intra‐ and inter‐assay coefficients of variation were 6.2% and 4.3%, respectively.

Since PV was expected to change in response to the intervention, and such changes could influence hormone concentrations independent of altered synthesis or clearance, circulating EPO content was also calculated by multiplying EPO concentration by PV at the corresponding time point.

### 2.6 Statistical analyses

All analyses were performed in GraphPad Prism 10 (GraphPad Software, Boston, MA, USA). Normality of continuous data was confirmed using the Shapiro–Wilk test and inspection of histograms and Q–Q plots; all variables met assumptions of normality.

Analyses were structured in two stages. First, to test the effect of the high‐altitude sojourn, haematological variables (Hb_mass_, RBCV, PV, BV, Hct, [Hb] and EPO) were analysed using two‐way repeated‐measures ANOVA (time × condition) between HA1 and HA14, enabling comparison of altitude‐related responses between CON and HWI groups. Second, to investigate the effect of the post‐altitude intervention, a subsequent two‐way repeated‐measures ANOVA was applied between HA14 and P5, with planned comparisons used to assess within‐group changes and between‐group differences at P5 (CON vs HWI).

Variables measured across the day of descent and the subsequent 24 h (EPO, TAMAX, [Hb]) were analysed separately as a distinct measurement window, using two‐way repeated‐measures ANOVA with planned comparisons to test between‐group differences at each time point. For all analyses, linear mixed models were applied in cases where missing data precluded a complete repeated‐measures structure.

Data are presented as the observed mean, and between‐condition differences as observed mean difference with 95% confidence intervals. Statistical significance was set at *P* < 0.05.

## 3 RESULTS

### 3.1 Haematological responses to high‐altitude sojourn

**FIGURE 2:**
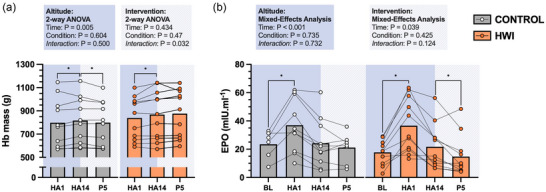
Haemoglobin mass (Hb_mass_; a) and circulating erythropoietin (EPO; b) responses to high‐altitude sojourn and subsequent post‐altitude intervention. Measurements were taken on day 1 (HA1) and day 14 (HA14) at 3800 m, and five days post‐descent (P5) at 1250 m, with baseline (BL) values included for EPO. Statistical comparisons shown above panels are derived from two separate analyses: (i) the altitude phase (HA1–HA14) and (ii) the post‐altitude intervention phase (HA14–P5), each analysed using two‐way repeated‐measures ANOVA (time × condition). For EPO, linear mixed models were applied where missing data precluded a complete repeated‐measures structure. *Significant within‐condition change between time points (*P* < 0.05). Data are presented as individual responses with group mean.

**FIGURE 3:**
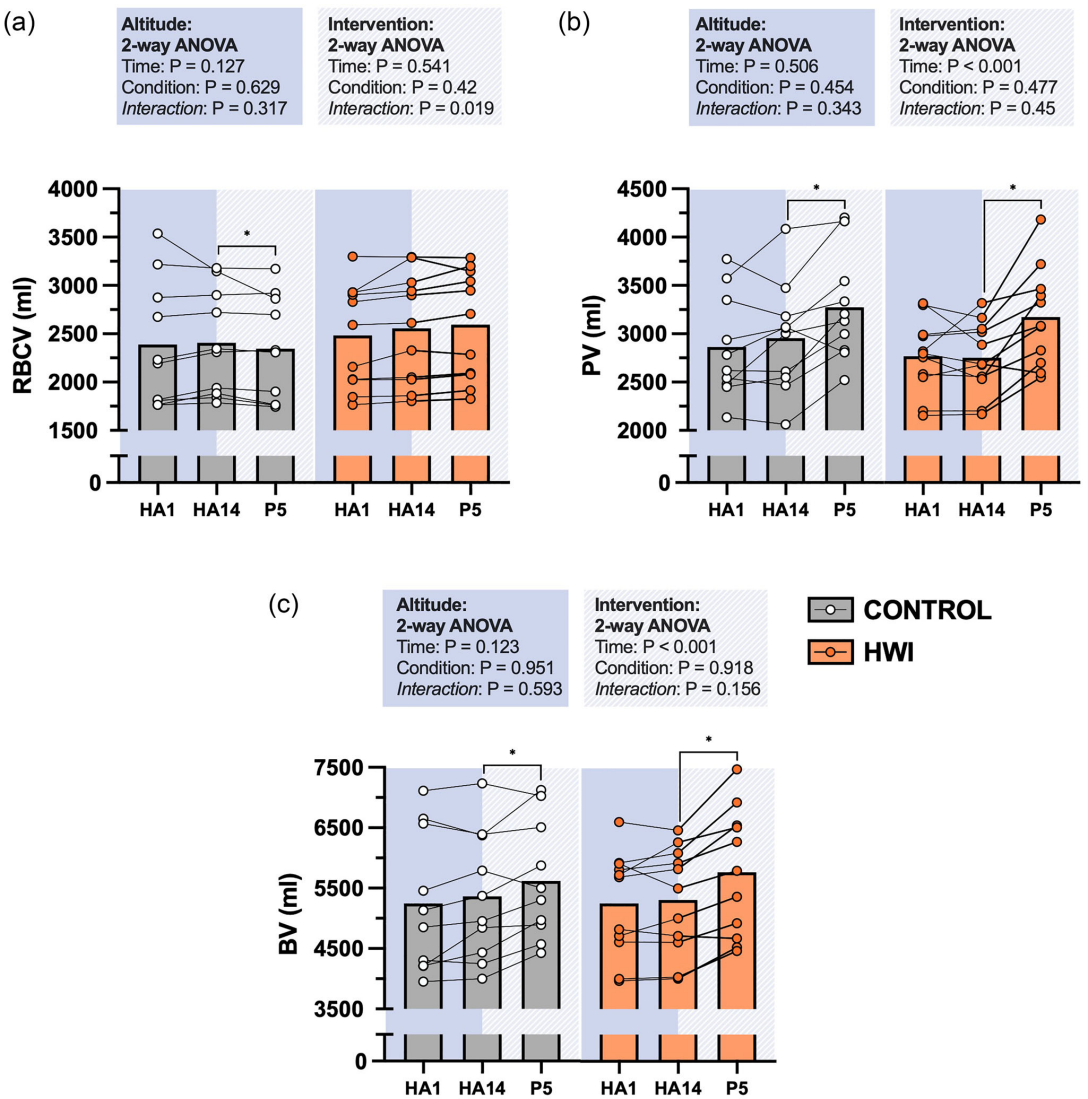
Red blood cell volume (RBCV; a), plasma volume (PV; b), and total blood volume (BV; c) responses to high‐altitude sojourn and subsequent post‐altitude intervention. Measurements were taken on day 1 (HA1) and day 14 (HA14) at 3800 m, and 5 days post‐descent (P5) at 1250 m. Statistical comparisons shown above panels are derived from two separate analyses: (i) the altitude phase (HA1–HA14) and (ii) the post‐altitude intervention phase (HA14–P5), each analysed using two‐way repeated‐measures ANOVA (time × condition). *Significant within‐condition change between time points (*P* < 0.05). Data are presented as individual responses with group mean.

Across all participants, Hb_mass_ increased by 24 g [95% CI: 8, 40] from HA1 to HA14 (*P* = 0.005; Figure 2a). This increase was similar in both groups (CON: ∆ = +19 g; HWI: ∆ = +29 g; *P* = 0.500) indicating comparable altitude‐induced responses prior to the intervention. Hb_mass_ at HA14 did not differ between CON and HWI (*P* = 0.563). RBCV did not change from HA1 to HA14 (∆ = +46 mL [−14, 106], *P* = 0.127; Figure 3a), with similar responses across groups (CON: ∆ = +16 mL; HWI: ∆ = +75 mL; *P* = 0.317). Likewise, PV (∆ = +38 mL [−79, 154], *P* = 0.506; Figure 3b) and BV (∆ = +89 mL [−27, 204], *P* = 0.123; Figure 3c) were unchanged over the altitude exposure, with no response differences between CON and HWI (all *P* ≥ 0.343). These findings indicate that any PV contraction likely occurred before the first CO‐rebreathe measurement (∼24 h after ascent). Consistent with these blood compartment responses, Hct (∆ = +0.2% [−1.0, 1.4], *P* = 0.751) and [Hb] (∆ = +0.2 g dL^−^^1^ [−0.2, 0.6], *P* = 0.412) did not change from HA1 to HA14, with no response differences observed between groups (all *P* ≥ 0.281).

Peripheral oxygen saturation ($S_{pO_{2}}$) increased by 3% [1.6, 4.4] from HA1 to HA14 (*P* < 0.001), averaging 93% at HA14. This was consistent across CON and HWI (*P* = 0.677).

Circulating EPO concentrations increased from BL to HA1 in both groups (CON: ∆ = +18 mIU mL^−^^1^ [2.6, 33.5], *P* = 0.028; HWI: ∆ = +21.3 mIU mL^−^^1^ [10.5, 32], *P* < 0.001; Figure 2b), before declining from HA1 to HA14 (CON: ∆ = −12.7 mIU mL^−^^1^ [−19.7, −5.7], *P* = 0.002; HWI: ∆ = −15 mIU mL^−^^1^ [−23.5, −6.5], *P* = 0.002). By HA14, EPO had returned to near‐baseline levels in both groups (CON: ∆ +3.5 mIU mL^−^^1^ [−15.6, 22.6], *P* = 0.826; HWI: ∆ +4.9 mIU mL^−^^1^ [−3.9, 13.6], *P* = 0.316). EPO concentrations did not differ between groups at any time point (all *P* ≥ 0.269).

### 3.2 Intervention efficacy: outcomes following descent

#### 3.2.1 Heat stress characteristics

All participants adhered well to the HWI protocol, with only one individual aborting their first session after 25 min due to discomfort. HWI elicited a consistent and robust thermal strain across the four monitored sessions (Table 2). Water temperature was tightly controlled (39.8 ± 0.2°C) and produced a 1.5 ± 0.4°C rise in tympanic temperature, reaching 38.5 ± 0.3°C at 45 min. This thermal load was accompanied by a sweat rate of 1.35 ± 0.5 L h^−^^1^ and was reflected in perceptual responses, with thermal sensation rising to 10.6 ± 0.9 and thermal discomfort to 6.3 ± 2.0 by the end of each session. Overall, the HWI protocol induced the intended physiological strain, driving marked elevations in core temperature, sweat loss and perceptual discomfort.

**TABLE 2:** Hot‐water immersion session characteristics.

	Tub temp (°C)	*T*_tymp_ @ 45 min (°C)	Δ*T*_tymp_ (°C)	Thermal sensation (1–13)	Thermal discomfort (1–10)	Sweat rate (L/h)	Tolerance (min)
Session 1	39.8 ± 0.4	38.9 ± 0.6	1.7 ± 0.5	10.8 ± 1.3	6.3 ± 2.4	1.15 ± 0.48	43.2 ± 6
Session 2	39.7 ± 0.2	38.6 ± 0.6	1.5 ± 0.7	10.5 ± 1	6.2 ± 2.2	1.35 ± 0.62	45 ± 0
Session 3	39.8 ± 0.2	38.3 ± 0.3	1.3 ± 0.5	10.5 ± 0.9	6.4 ± 2.1	1.44 ± 0.49	45 ± 0
Session 4	39.7 ± 0.1	38.3 ± 0.4	1.5 ± 0.6	10.4 ± 1.3	6 ± 2.2	1.46 ± 0.47	45 ± 0
Mean	39.8 ± 0.2	38.5 ± 0.3	1.5 ± 0.4	10.6 ± 0.9	6.3 ± 2	1.35 ± 0.5	44.5 ± 1.5

*Note*: Data are means ± SD. *T*_tymp_ @ 45 min, tympanic temperature at 45 min of hot‐water immersion session; Δ*T*_tymp_, change in tympanic temperature across 45 min hot‐water immersion. Thermal sensation (1 = unbearably cold, 13 = unbearably hot) and thermal discomfort (1 = comfortable, 10 = extremely uncomfortable) were rated using scales adapted from Gagge et al. (1967) and presented values were recorded immediately prior to tub exit.

#### 3.2.2 Day of descent: Subsequent 24‐h responses

**FIGURE 4:**
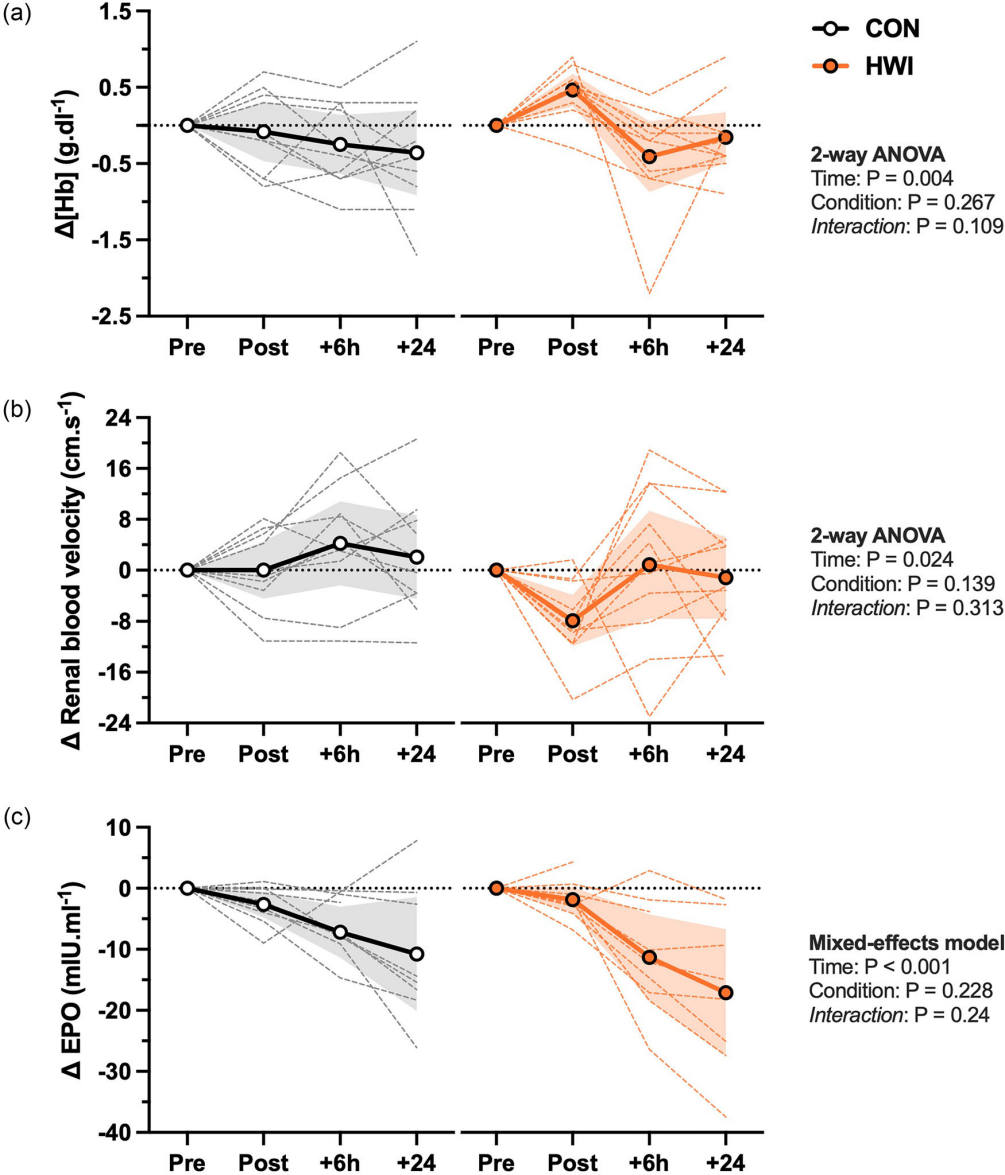
Haemoglobin concentration ([Hb]; a), renal artery blood velocity (time‐averaged maximum; TAMAX; b), and circulating erythropoietin (EPO; c) responses over the 24‐h sampling cycle on the day of descent. Measurements were obtained before (Pre) and immediately after (Post) the first hot‐water immersion (HWI) or time‐matched control (CON) session, with additional samples collected at +6 h and +24 h. Statistical comparisons shown are derived from two‐way repeated‐measures ANOVA (time × condition). For EPO, linear mixed models were applied where missing data precluded a complete repeated‐measures structure. Data are presented as individual responses with group mean ± 95% CI.

Following descent to low altitude, [Hb] decreased from Pre to +24 h (*P* = 0.004; Figure 4a). Although there was no time × condition interaction (*P* = 0.109), the change in [Hb] post‐immersion was 0.5 g dL^−^^1^ [0.1, 1.0] higher in HWI than CON (*P* = 0.015), consistent with an expected acute PV contraction. Groups did not differ at +6 h or +24 h time points (*P* ≥ 0.487).

Renal artery blood velocity changed across the sampling period (*P* = 0.024; Figure 4b). Despite no time × condition interaction (*P* = 0.313), the change in TAMAX post‐immersion was 7.9 cm s^−^^1^ [−14.8, −1.0] lower in HWI than in CON (*P* = 0.025). Values were similar between groups at +6 h and +24 h time points (*P* ≥ 0.336).

EPO concentrations declined following descent (*P* < 0.001; Figure 4c), with no difference between groups (*P* = 0.228), and no time × condition interaction (*P* = 0.24). From pre to +24 h, EPO decreased by 10.7 mIU mL^−^^1^ [−18.2, −3.2] in CON (*P* = 0.002) and by 16.8 mIU mL^−^^1^ [−24.2, −9.4] in HWI (*P* < 0.001). These findings were unchanged when EPO was expressed as circulating content, accounting for transient PV shifts (Table 3).

**TABLE 3:** Total EPO content (IU) responses to high‐altitude sojourn and post‐altitude intervention.

	High‐altitude sojourn		Sampling cycle on day of descent				Post‐altitude
	HA1	HA14	Pre	Post	+6 h	+24 h	P5
CON	105.5 ± 50.8	72.7 ± 51.8	83.1 ± 57.6	75 ± 53.9	67.1 ± 46.1	67.5 ± 54.6	73.4 ± 37
HWI	100.4 ± 50	58.1 ± 41.2	59.7 ± 49.8	54.9 ± 42.1	37.9 ± 24.2	24.2 ± 17.5	48.1 ± 48.8

*Note*: Data are means ± SD. Time points are defined as follows: HA1, high‐altitude day 1; HA14, high‐altitude day 14; Pre, day of descent, immediately before the first hot‐water immersion (or time‐matched control); Post, +6 h and +24 h, immediately after, 6 h after and 24 h after the first immersion; P5, post‐descent day 5. EPO content was calculated as serum EPO concentration (mIU mL^−^^1^) × plasma volume (mL) and expressed in international units (IU = mIU/1000). For the sampling cycle conducted on the day of descent, plasma volume values were estimated using the Dill & Costill (1974) method based on changes in haematocrit and haemoglobin concentration.

Statistical summary: EPO content declined over the high‐altitude sojourn (*P* < 0.001) with no between‐group difference (*P* = 0.635). Across the day of descent, EPO content decreased with time (*P* < 0.001), but did not differ between groups (*P* = 0.137; time × condition *P* = 0.487). During the post‐altitude intervention period, no effects of time (*P* = 0.619), condition (*P* = 0.245), or their interaction (*P* = 0.153) were detected.

#### 3.2.3 Haematological consequences of hot‐water immersion

Hb_mass_ responded differently between groups (time × condition interaction: *P* = 0.032; Figure 2a). From HA14 to P5, Hb_mass_ decreased by 18 g [−36, 0] in CON (*P *= 0.045) but remained unchanged in HWI (+9 g [−8, 26]; *P* = 0.285), resulting in a between‐group difference of 27 g [10, 44] (*P* = 0.002). A similar pattern was observed for RBCV (time × condition: *P* = 0.019; Figure 3a). RBCV declined by 59 mL [−115, −3] in CON (*P* = 0.041) but was preserved in HWI (∆ = +36 mL [−18, 90], *P* = 0.177), yielding a between‐group difference of 95 mL [42, 148] (*P* < 0.001). PV increased following descent (∆ = +371 mL [228, 513], *P* < 0.001; Figure 3b), with similar expansion in CON and HWI (*P *= 0.45). BV also increased (∆ = +358 mL [214, 501], *P* < 0.001), again with no difference between groups (*P* = 0.156), although BV at P5 was 203 mL [6, 400] higher in HWI than CON (*P* = 0.044). Hct decreased by 3.2% [−4.4, −1.9] during the intervention period (*P* < 0.001), while [Hb] decreased by 1.1 g dL^−^^1^ [−1.5, −0.6] (*P* < 0.001), with no differences between groups (all *P* ≥ 0.903).

$S_{pO_{2}}$ increased by 4.7% [3.7, 5.7] 5 days after return to 1250 m (*P* < 0.001), with no difference between groups (*P* = 0.423).

EPO decreased by 4.0 mIU mL^−^^1^ [−7.8, −0.2] from HA14 to P5 (*P *= 0.039; Figure 2b). There were no group differences in response (*P* = 0.124), and P5 concentrations were not different between CON and HWI (*P* = 0.239). Circulating EPO content showed no main effects or interaction (all *P ≥* 0.153; Table 3).

## 4 DISCUSSION

This study demonstrates the capacity of HWI to maintain an altitude‐induced rise in Hb_mass_ across the first 5 days following descent. Although PV expanded similarly in both groups, differences in RBCV retention produced a greater total BV in HWI at P5. A single session of HWI induced a transient haemoconcentration that, contrary to expectations, was not followed by measurable PV expansion at +6 or +24 h post immersion. HWI also produced a reduction in renal artery blood velocity, which normalised within 6 h. Despite these acute responses, post‐altitude EPO concentrations declined to a similar extent across the first 24 h in both groups and were not higher in HWI compared to CON at P5, suggesting that maintenance of Hb_mass_ may not have required sustained EPO elevation. Collectively, these findings indicate that brief, repeated HWI during deacclimitisation can sustain the haematological benefits of altitude, and may thereby extend the window of performance advantage for athletes competing following altitude training.

### 4.1 Passive heat stress sustains the haematological benefits of altitude exposure

This study is the first to demonstrate that *passive* heat acclimation can be utilised to maintain post‐altitude haematological adaptations, in line with recent findings from Rønnestad et al. (2024) using *active* heat‐suit training. That HWI was sufficient to maintain Hb_mass_ following deacclimitisation is consistent with our earlier work showing that 5 weeks of HWI can increase Hb_mass_ to a similar extent as both active heat acclimation protocols and traditional altitude training (Jenkins et al., 2025b). These findings extend prior observations by showing that passive heat stress can not only induce haematological adaptation, but also prevent its rapid regression following altitude descent (Klein et al., 2021; Mairbäurl, 2018; Merino, 1950).

From an applied perspective, passive heat acclimation is particularly valuable. Unlike active heat training, HWI does not compromise training intensity, increase mechanical load or heighten inflammation (Menzies et al., 2025; Nybo & Nielsen, 2001; Periard et al., 2021; Steward et al., 2025), enabling athletes to resume optimal training practices upon return to sea level. This may be especially important when training intensities have already been constrained by hypoxia during altitude residence (Levine & Stray‐Gundersen, 1997; Saunders et al., 2009). Preserving altitude‐derived haematological benefits without imposing additional training strain therefore provides a practical athlete load‐management strategy during the critical taper phase preceding competition.

The simplicity of HWI further enhances its applied relevance, requiring minimal equipment and allowing implementation within typical training settings (Heathcote et al., 2018; Jenkins et al., 2025a). While our study involved a recreationally active, mixed‐sex cohort, the complementary findings of Rønnestad et al. (2024) in elite cyclists suggest that the approach may be applicable across training backgrounds. Collectively, these data highlight HWI as a feasible and effective method to sustain altitude‐induced Hb_mass_ benefits in the weeks leading into competition.

### 4.2 Underlying mechanisms of heat‐mediated Hb_mass_ preservation

A notable feature of the present study was that Hb_mass_ was maintained in HWI but declined in CON, despite no differences in circulating EPO between groups. In line with prior work, we observed a marked decline in EPO following withdrawal of the hypoxic stimulus (Płoszczyca et al., 2018). It could have been expected that Hb_mass_ retention upon descent would be supported by acute heat‐induced EPO elevations, since DiMarco et al. (2024) demonstrated that a single bout of HWI reduced renal artery blood velocity and, in turn, increased circulating EPO within 6 h. While we similarly observed a transient fall in renal artery blood velocity following HWI, this was not accompanied by a rise in EPO at +6 h, despite the matching of HWI conditions (40°C for 45 min; DiMarco et al. 2024). Although our data do not support a role for EPO in Hb_mass_ preservation following HWI, this discrepancy may reflect residual elevation of endogenous EPO persisting from high‐altitude exposure on the first day of descent, which could have obscured any additional heat‐induced response. By P5, the final blood sample was drawn ∼24 h after the last immersion, by which point any transient EPO response may have resolved.

Even if such short‐lived EPO elevations were missed, the ‘critmeter’ mechanism, whereby PV expansion dilutes Hct and triggers compensatory EPO release (Donnelly, 2001), is unlikely to explain our findings. Hct decreased to a similar extent from HA14 to P5 in both groups, making differential volume shifts an improbable driver of Hb_mass_ preservation. Instead, our findings resemble endurance training interventions in which haematological adaptation continues despite normalisation of circulating EPO (Montero & Lundby, 2018; Montero et al., 2017). In untrained adults undergoing 8 weeks of supervised endurance training, EPO rose transiently at week 2, and subsequent increases in Hb_mass_ (+10 % by week 8) occurred after EPO had returned to baseline (Montero et al., 2017). Across that intervention, RBCV was inversely associated with EPO, suggesting that once erythropoiesis is initiated, it may proceed independently of sustained EPO elevation. This pattern points to additional endocrine factors that may sustain erythropoiesis in the absence of chronically elevated EPO. One potential, albeit speculative, candidate is cortisol, given its capacity to promote progenitor cell differentiation and mobilisation (Hanssen & Iskander, 2025; Peschle et al., 1971; Varricchio et al., 2022). As with exercise (Hill et al., 2008), heat stress is also a robust stimulus for cortisol release, both during exercise in the heat (Costello et al., 2018; Pilch et al., 2022) and passive exposures (Park et al., 2021). Repeated cortisol elevations during HWI may therefore have provided a permissive environment for continued erythropoiesis, even without sustained increases in circulating EPO.

An additional observation was that Hb_mass_ declined more rapidly in CON than typically reported, as previous studies suggest regression toward baseline over 1–2 weeks rather than within 5 days (Klein et al., 2021; Mairbäurl, 2018; Ryan et al., 2014; Siebenmann et al., 2015). This accelerated drop may reflect the relatively short altitude exposure in our study (14 days at 3800 m), during which Hb_mass_ increased by 2.4% (19 g) in CON. By comparison, Klein et al. (2021) reported a 4.7% increase after a 3‐week sojourn (3450 m), and meta‐analytic modelling by Gore et al. (2013) predicts an increase of ∼6% after 4 weeks (range of 1360–3600 m), suggesting that the magnitude of Hb_mass_ expansion may influence subsequent decay. Alternatively, cohort‐specific factors (e.g., sex distribution, training status, iron availability) may also have contributed to the decrease. Ultimately, the accelerated decline in CON underscores the contrast with HWI, where Hb_mass_ was maintained.

Together, these observations provide a physiological precedent that Hb_mass_ can be maintained post‐altitude in the absence of sustained EPO elevation. Small or short‐lived EPO perturbations not captured within the present sampling window could have been sufficient to stabilise erythropoiesis, with additional endocrine signals such as cortisol potentially contributing, though this remains speculative and warrants further investigation.

### 4.3 Limitations

Several limitations should be acknowledged when interpreting our findings. First, although HWI preserved Hb_mass_ following altitude exposure, our cohort comprised recreationally active individuals studied at 3800 m for 14 days. Elite athletes, by contrast, typically conduct training camps at more moderate altitudes of ∼1800–2500 m for periods of 3–4 weeks (Girard et al., 2023; Saunders et al., 2009), which may limit the applicability of our findings into competitive practice. Second, while participants were broadly activity‐matched, training status (e.g., ${\dot{V}}_{O_{2}\max}$) was not assessed, and fitness levels can influence the magnitude and timing of endocrine responses to stress, with more highly trained individuals showing distinct EPO and hormonal dynamics (Bodary et al., 1999; Montero et al., 2017). Third, we employed a time‐matched control rather than a thermoneutral immersion condition. Water immersion, irrespective of temperature, introduces hydrostatic pressure, which can acutely elevate EPO (Chudek et al., 1997). Consistent with a possible role of immersion, Hb_mass_ has been reported to be maintained up to 10 days following altitude exposure in elite swimmers (Carin et al., 2025), a population characterised by substantial daily exposure to water immersion. A thermoneutral comparator would have more precisely isolated hydrostatic versus thermal influences on haematological outcomes; however, our intention was to evaluate repeated passive heat exposure as a practical intervention, and a time‐control was considered the most relevant comparator for applied settings. Fourth, the environmental conditions at our low altitude testing site in Owens Valley (36°C average daily high, 15% relative humidity) may have contributed to the PV expansion observed in both groups; however, the absence of Hb_mass_ preservation in CON emphasises the efficacy of HWI. Fifth, iron status (i.e., serum ferritin) was not assessed or supplemented, and insufficient iron availability could have constrained erythropoietic responses in some individuals (Okazaki et al., 2019). Finally, although clear haematological benefits were observed, performance outcomes were not measured, leaving open the question of whether post‐altitude HWI translates into extended performance advantages at sea level.

### 4.4 Conclusion

This study tested whether passive heat exposure by HWI in the days following altitude descent could sustain haematological adaptations. Specifically, we evaluated the effects of repeated HWI on Hb_mass_, BV, EPO and renal haemodynamics. The intervention maintained the altitude‐induced rise in Hb_mass_ and, alongside comparable PV expansion to controls, produced a greater total BV at P5. Acute responses to HWI included transient haemoconcentration and reduced renal artery blood velocity, both of which normalised within hours and were not accompanied by elevations in EPO. Collectively, these findings demonstrate that passive heat exposure during deacclimitisation can help retain altitude‐induced Hb_mass_ expansion, providing new insight into strategies that may modulate the decay of haematological adaptations after hypoxic exposure.

## AUTHOR CONTRIBUTIONS

Elliott J. Jenkins and Mike Stembridge conceived the research. Elliott J. Jenkins, Michael G. Hughes, Joshua C. Tremblay and Mike Stembridge contributed to the study design. Elliott J. Jenkins, Jodie L. Koep, Andrew J. M. Douglas, Lauren E. Maier, Connor A. Howe, Sarah Sheitelman, Liam D. Corr, Christoph Siebenmann, Joshua C. Tremblay, Phillip N. Ainslie, Travis D. Gibbons and Mike Stembridge collected the data. Elliott J. Jenkins performed the data analysis and interpreted the data with input from Michael G. Hughes, Joshua C. Tremblay and Mike Stembridge. Elliott J. Jenkins drafted the manuscript. All authors critically revised the manuscript, approved the final version, and agree to be accountable for all aspects of the work, ensuring that questions related to the accuracy or integrity of any part are appropriately investigated and resolved. All persons designated as authors qualify for authorship, and all those who qualify for authorship are listed.

## CONFLICT OF INTEREST

None declared.

## FUNDING INFORMATION

None.

## Data Availability

The datasets generated and analysed during the current study are available from the corresponding author upon reasonable request.
